# Transforming health policy through machine learning

**DOI:** 10.1371/journal.pmed.1002692

**Published:** 2018-11-13

**Authors:** Hutan Ashrafian, Ara Darzi

**Affiliations:** Institute of Global Health Innovation (IGHI), Imperial College London, London, United Kingdom

## Abstract

In their Perspective, Ara Darzi and Hutan Ashrafian give us a tour of the future policymaker's machine learning toolkit.

Machine learning (ML) is one of the most prominent applications of artificial intelligence (AI) technology and offers multiple routes to support the core objectives of health policy. These include ‘creating the conditions that ensure good health’ [1] and social care for an entire population through preventive strategies, protection from disease, promotion of healthy lifestyles, and population screening through knowledge capture (typically in the form of big data). Overall governance will offer a patient-centred approach with the consideration of patient advocacy and workforce and resource management [2] (Fig 1). Herein, we will break down the role of ML in each of these areas.

**Fig 1:**
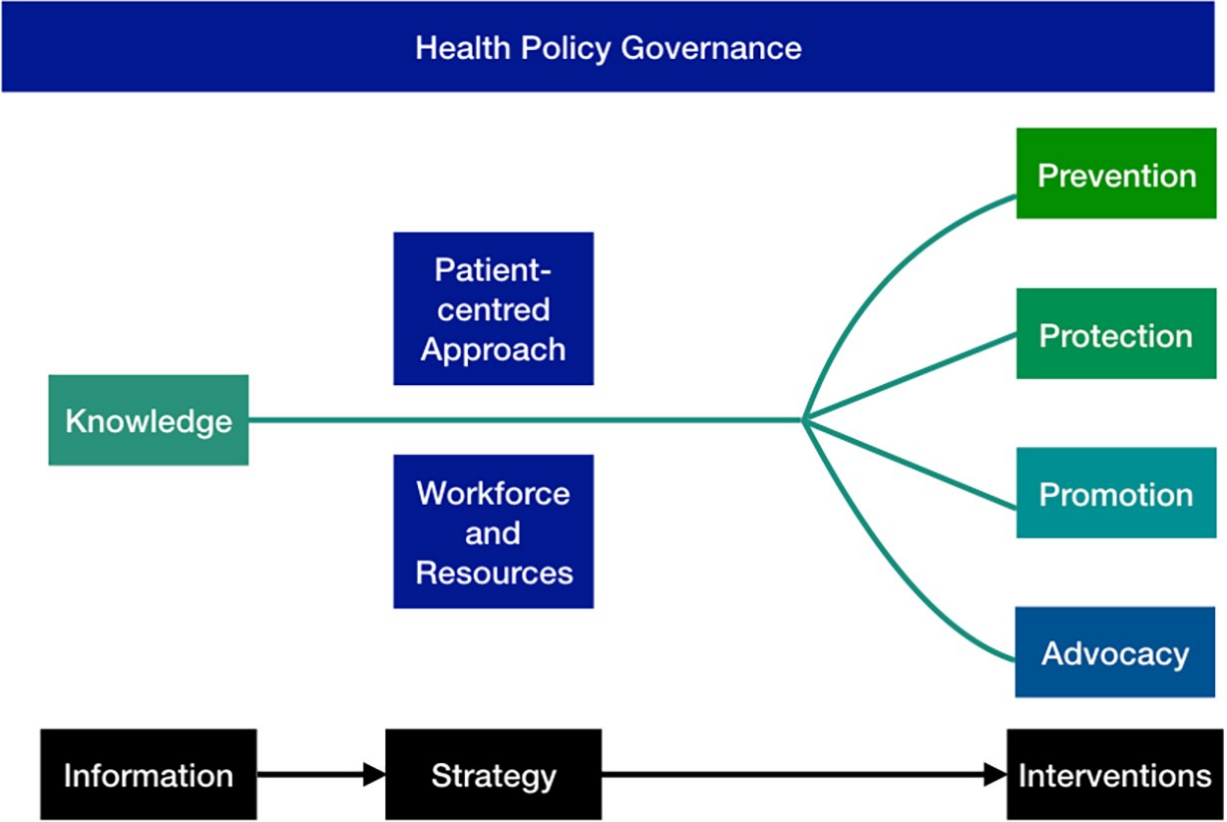
The components of health policy.

The most prominent contribution of AI to health policy knowledge currently resides within the application of ML to large, population-level datasets such as those from medical imaging, electronic health records (EHRs), and whole-genome studies. This information can guide interventions for high-risk individuals. Current applications can outperform established risk scores to predict clinical outcomes. These include in-hospital mortality, 30-day unplanned readmission, prolonged length of stay, and final discharge diagnoses for patient populations numbering in the several hundred thousands [3]. Here, the major limitation is access to large and high-quality population-level datasets with which to apply ML approaches. We feel that the unification of the United Kingdom National Health Service (NHS) dataset of over 66 million individuals in the form of EHRs or even patient health records (PHRs; in which patient data accompany the patients directly) can offer one of the largest datasets worldwide for analysis by ML. This could offer improved predictions for clinical outcomes from current records and could also provide novel hypothesis-generating concepts that may lead research to better understand disease behaviours and their treatments.

Enhanced population protection can also be derived from ML analysis of data from a variety of digital sources to better predict population-level disease diffusion through ‘crowd surveillance’. Here, social media data (such as tweets) can monitor the spread of influenza in order to arrange timely vaccine supplies or alternatively understand the reasons and sentiments behind the low vaccine coverage [4] so that strategies to enhance uptake can be implemented. They can also be used to identify the illegal online sale of prescription opioids from global online vendors [5]. Environmental data from climate sensors can predict climate crises, ecosystem shifts, and pollution trends [6] with higher accuracy than current systems to allow preparation for emergency responses to climate situations or support ecological management strategies. Data from city transportation audits or integrated smart city sensors can also be applied to predict locations of injuries or trauma due to car crashes within towns and cities [7] and inform site-specific interventions to prevent urban vehicular accidents.

The application of ML in modern marketing strategies also highlights the potential for this technology in health promotion. The next generation of recommender systems (information-filtering systems that support decisions) can guide health behaviours. Here, the application of ML algorithms have been identified as offering powerful solutions to content-based analysis (based on personality information), collaborative filtering (in which past behaviour is utilised to predict future actions), or hybrid approaches (using both techniques) to assess online behaviours [8]. The information for this can be obtained from EHRs with the consent and national regulatory adherence in order to target unhealthy behaviours such as buying tobacco-based products or the individual purchasing of alcohol- or sugar-based drinks.

ML-based population screening will offer new inroads into disease protection through enhanced screening strategies. Here, ML flagging systems have been applied to identify patients at a high risk for colorectal cancer based on a simple complete blood count test [9]. This test was utilised to help identify individuals at high risk of colorectal cancer who were noncompliant to a national screening programme; however, this technology may eventually have the capacity to offer full population screening (also allowing for personalised screening). Deep-learning image screening, for example, on mammography is currently being developed and has the potential to enhance health delivery by supporting scalable, cost-effective diagnostic decisions.

Patient advocacy ensures the continued application of ethics as a recognised contributor to population well-being. The increased application of big-data analytics [10] can result in the reinforcement of historical biases (and therefore discrimination), for example, in declining the opportunity of health insurance to individuals of a particular race/ethnic group or demographic because of an unrepresentative or biased data source. However, if applied judiciously, ML approaches have the potential to assess for disparities or unjustified data discrimination to ensure adherence to accepted guidelines and data health justice across society.

In terms of resources or capacity, ML has the potential to help address massive healthcare practitioner shortages worldwide. Initially, this would likely take the form of supporting diagnostic activity but would also play an increasing role in all stages of health and care, ranging from booking appointments for health staff, supporting interventional decisions, and eventually offering direct prescriptive healthcare advice [11]. This prospect has initiated a formidable societal controversy, as arguments over the benefits of AI in supporting resource deficits have also been countered with arguments that AI will lead to massive job losses—for example, in diagnostic radiology or pathology, for which an ML algorithm could appraise multitudes of images on a 24-hour work cycle. Many of these issues carry an impact beyond that of healthcare and have a bearing on national and international economic strategy as well as the wider public discourse on the exact role of AI in society. We suggest that AI’s first and least perilous role should be in resourcing healthcare. This will likely disrupt current work practices but will also generate new jobs and roles. More importantly, ML-based technologies may offer society ‘freed-up’ health practitioner time to focus on direct patient care.

The transformative potential of ML in health and care policy supports the prediction of Alan Turing (1950) that AI, or ‘machine intelligence’ [12], will have a widespread and pervasive role within our society. Arguably the least explored area of AI in health policy is its role in governance, which ranges from legislation to strategy, financing, and accountability. Here, ML solutions could offer rapidly produced analytics and appraisal of policy statements. Using these, policy makers and politicians can drive the next generation of health policies. Although many of these ML systems remain experimental and theoretical, they could ultimately present the largest transformative role in health governance to date.

## Abbreviations

AI: artificial intelligence
EHR: electronic health record
ML: machine learning
NHS: National Health Service
PHR: patient health record
